# The efficacy of Phoslock® in reducing internal phosphate loading varies with bottom water oxygenation

**DOI:** 10.1016/j.wroa.2021.100095

**Published:** 2021-03-05

**Authors:** Mary A. Zeller, Marc J. Alperin

**Affiliations:** a Geochemistry and Isotope BioGeoChemistry Group, Department of Marine Geology, Leibniz Institute for Baltic Sea Research (IOW), 18119 Warnemünde, Germany; b Department of Marine Sciences, University of North Carolina, Chapel Hill, North Carolina, United States

**Keywords:** Phoslock®, Phosphate flux, Nutrient management, Core incubations, Oxia, Anoxia, Iron-rich sediments

## Abstract

Eutrophication in lakes and reservoirs has prompted interest in using sediment capping technology to reduce the sediment contribution to internal nutrient loading. One such sediment capping technology is Phoslock®, a lanthanum-embedded clay, which can bind phosphate at the sediment surface and limit its diffusion into the water column. However, in well-oxygenated lakes, naturally occurring iron can bind phosphate by a similar mechanism. We sought to test the efficacy of Phoslock® in limiting phosphate (PO_4_^3−^) fluxes relative to untreated iron-rich lake sediment under conditions of bottom-water oxia and anoxia through laboratory batch core incubations of intact sediment cores from Jordan Lake, a reservoir in central North Carolina. We found that Phoslock® decreased phosphate fluxes relative to the control under anoxic conditions (7.5 ± 9.5 vs. 236 ± 74 µmol PO_4_^3−^•m^−2^•d^−1^), but provided no benefit relative to the control when the water column was oxygenated (4.5 ± 4.3 vs. 7.0 ± 11.4 µmol PO_4_^3−^•m^−2^•d^−1^). We also found that Phoslock® itself can act as a source of NH_4_^+^ to Jordan Lake waters. Applied at recommended levels to the whole lake, Phoslock® addition would result in a pulse increase in water column NH_4_^+^ concentrations of approximately 2.6 ± 0.8 μM (an increase of 10 to 275% compared to ambient).

## Highlights

• Phoslock® decreases iron-rich sediment-water P flux when bottom waters are anoxic.
• Phoslock® does not decrease sediment-water P flux when bottom waters are oxic.
• Phoslock® is a source of N—NH_4_^+^ to the water column when dispersed in lake water.

## Graphic Abstract

**Figure:**
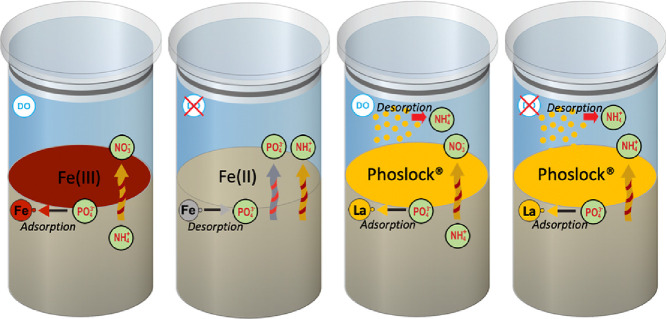

## 1 Introduction

Nutrient loading, including both nitrogen (N) and phosphorus (P), is responsible for widespread and long-term eutrophication in lakes, a process that is increasing in magnitude as humans continue to affect freshwater ecosystems through agriculture and development (Dodds et al., 2013; Smith et al., 2006; Wurtsbaugh et al., 2019). Once nutrients enter a freshwater lake or reservoir from the watershed, they can stimulate primary productivity and harmful algal blooms, and enhance the delivery of organic matter to the sediments (Le Moal et al., 2019). Here, various remineralization processes regenerate nutrients that accumulate in the porewater and can be returned to the water column through processes of diffusion (Berg et al., 1998), bioirrigation (Murniati et al., 2017; Renz et al., 2018), and bioturbation (Berg et al., 2001). As a result of this benthic recycling (often referred to as ‘internal nutrient loading’), nutrients that enter a lake can contribute to eutrophication for many years (Søndergaard et al., 2013).

Strategies for coping with nitrogen loading often rely on the establishment of denitrification-favorable conditions (Vymazal, 2007), aimed at permanent removal of reactive N from the environment by the biological reduction to non-reactive N_2_ gas. However, managers hoping to reduce or reverse eutrophication in lakes often focus on reducing phosphorus loading (Schindler et al., 2008, 2016), which increases the N:P ratio, potentially limiting the dominance of N_2_-fixing cyanobacteria that can be a health hazard and nuisance (Schindler, 2012). Still, the concept that P-loading alone can solve lake eutrophication is controversial (Paerl et al., 2011), due to the negative impacts that excess N can have on lake macrophyte communities (Moss et al., 2013). Additionally, managed reductions of P in lakes without accompanying N management can decrease the ability of lakes to remove reactive N through nitrification/denitrification (Finlay et al., 2013), and exacerbate downstream eutrophication in often N-limited estuaries and coastal areas (Paerl, 2009).

In some lakes, especially those with clay-rich sediments, iron can effectively ‘cap’ the release of phosphate from the sediments when the water column is oxygenated, through the redox-dependent formation of Fe(III)-phosphate complexes in the oxygenated layers of sediment (Orihel et al., 2015, 2016). Additionally, lake sediments with low sulfide and high iron can permanently sequester P, through the formation of the stable mineral vivianite in anoxic sediments, eventually reducing concentrations of leachable P and thus its release to the water column (Gächter and Müller, 2003; Rothe et al., 2014). When these natural processes are insufficient sinks for P loading, and/or it is not practical to decrease P loading from the watershed, stakeholders may increasingly turn to engineering solutions, such as sediment capping or dredging, to decrease internal P loading (Lürling et al., 2017; Lürling and Faassen, 2012; Guido Waajen et al., 2016; Zamparas and Zacharias, 2014). One such approach is the capping of sediment with lanthanum-embedded bentonite clay, known commercially as Phoslock®.

Phoslock® has already been applied to a variety of lakes worldwide, across broad ranges in morphology and designated use (Bishop et al., 2014; Bishop and Richardson, 2018; Copetti et al., 2015; Dithmer et al., 2016b; Spears et al., 2013, 2016). While these studies generally show a reduction in water column soluble reactive phosphorus (SRP), other studies have highlighted unintended impacts of Phoslock® on sediment biogeochemistry, such as changing the location of the sediment oxic-anoxic boundary (Vopel et al., 2008). Additional risks include ecological toxicity, related to the leaching of lanthanum from Phoslock®, especially in lakes of low alkalinity (Gibbs et al., 2011; Reitzel et al., 2017; Spears et al., 2013), and associated bioaccumulation of lanthanum in the ecosystem has been reported (Van Oosterhout et al., 2014; G. Waajen et al., 2017).

Other studies have indicated that Phoslock® may act as a direct source of ammonium (NH_4_^+^) when leached with ultrapure water in the laboratory, including nanopore (van Oosterhout and Lürling, 2013) and Milli-Q (Reitzel et al., 2013) water. This ammonium may come from the bentonite clay matrix itself (Hanway et al., 1957) and may not be removed during the Phoslock® manufacturing process. While studies have shown that the Phoslock® clay matrix can be unstable under particular natural lake conditions, such as low alkalinity and/or high humic substance content, as demonstrated by the release of La (Reitzel et al., 2017; Spears et al., 2013), studies have yet to demonstrate whether NH_4_^+^ is leached from Phoslock® in natural lake water. Furthermore, evidence is presently lacking for the efficacy of Phoslock® on intact sediment-water interfaces, as opposed to homogenized surface sediment (Egemose et al., 2010; Gibbs et al., 2011; Reitzel et al., 2013; Wang et al., 2016), where its effects on sediment biogeochemistry are expected to be more representative of the natural lake bed. Core incubation studies have demonstrated that Phoslock® amendment can decrease the release of P from homogenized sediments from Lake Rotorua (Gibbs et al., 2011) and Lake Langesø (Reitzel et al., 2013) relative to untreated sediment, even under oxic conditions when surface iron(III) oxides would be potentially available to bind P. It is probable that the relative efficacy of Phoslock® is dependent on characteristics of particular lakes, such as the iron content in the sediments, and we were interested in testing this relative efficacy for clay-rich lake sediments common to the southeastern United States.

In this study, we tested the relative efficacy of Phoslock® in reducing benthic phosphate fluxes compared to untreated iron-rich cores under conditions of bottom-water oxia and anoxia. This study took place in a shallow reservoir in central North Carolina, representative of Piedmont, clay-rich reservoirs throughout the southeastern United States. We used batch incubations of intact sediment cores to measure the impact of Phoslock® on sediment-water phosphate fluxes under conditions of oxia and anoxia, and also observed whether ammonium is leached from Phoslock® with natural lake water.

## 2 Methods

### 2.1 Study site

**Fig. 1:**
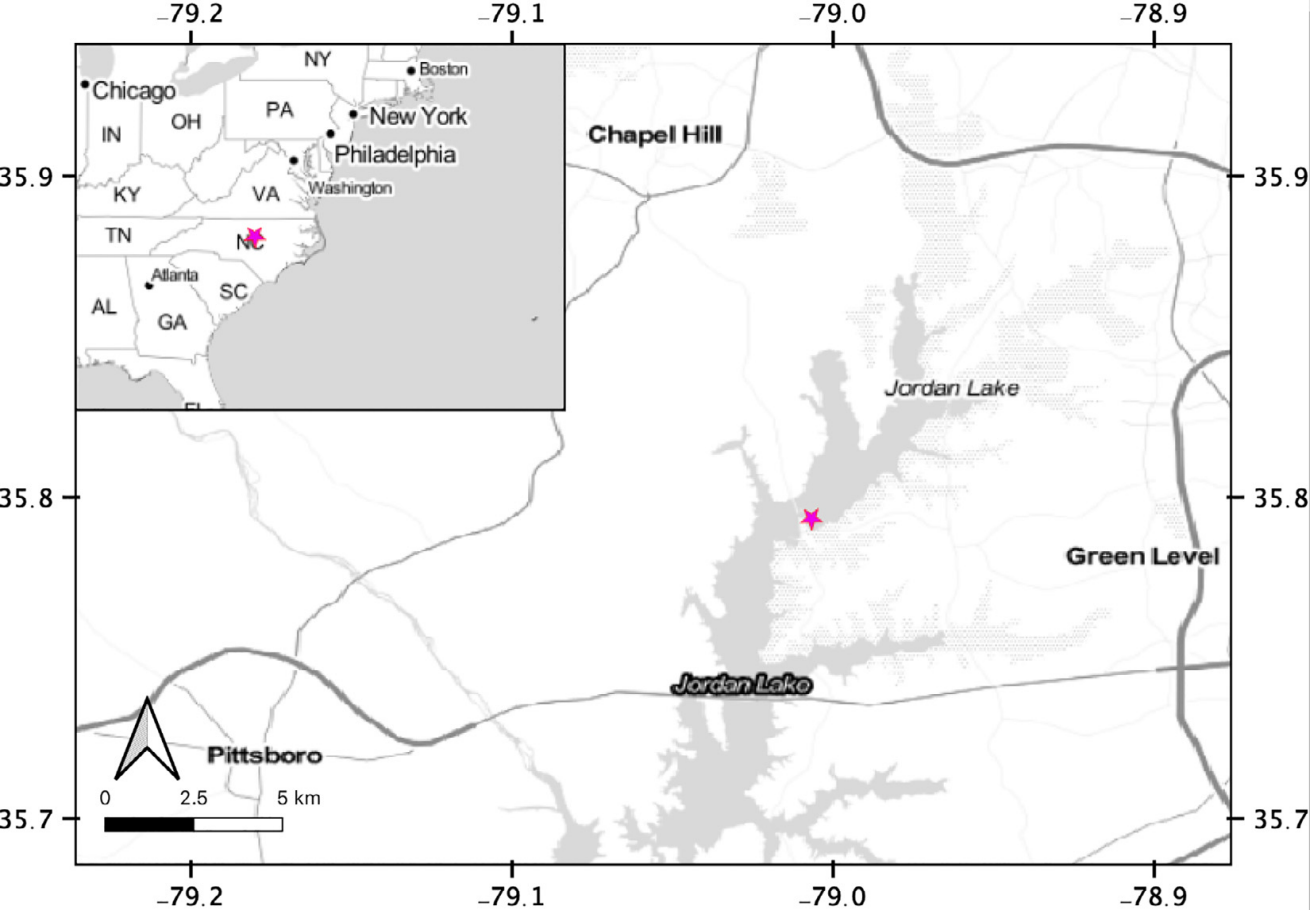
Map of our site (pink star) within Jordan Lake, and within the eastern United States (inset). All three sampling campaigns were conducted in the same area of Jordan Lake.

B. Everett Jordan Lake (Fig. 1) is a reservoir in central North Carolina that provides drinking water for the towns of Cary, Apex and Morrisville, and Chatham and Wake Counties, as well as recreation and other services to the broader Triangle region (Berke et al., 2013). The reservoir was constructed from 1973 to 1983 with the damming of the Haw and New Hope rivers, within the Cape Fear River drainage basin. Jordan Lake has a long history of impaired water quality, frequently failing to meet United States Environmental Protection Agency (US EPA) guidelines for Chlorophyll a, along with year-round detectable concentrations of microcystin and anatoxin (Wiltsie et al., 2018). While Jordan Lake is shallow (average depth 4.3 m, max 12 m) and generally well-mixed, some sections experience stratification and bottom-water anoxia during the summer (Cain, 2017).

### 2.2 Sampling strategy

Short sediment cores (~15–30 cm in length, with core length determined by the availability of sediment above the historic riverbed) were collected using a Kajak-Brinkhurst (KB) corer with 4.7 cm (id) core liner (Wildco Supply Company). All cores were taken near the North Carolina Division of Water Resources station CPF086F, northeast of the State Road 1008 bridge in Jordan Lake (Fig. 1), between October 2017 and April 2018. In October 2017, 8 cores were collected, with 4 designated for use in the flux experiments and 4 sectioned immediately for pore-water and solid-phase analysis. In February 2018, 8 cores were again collected (5 for flux experiments and 3 for immediate sectioning), while in April 2018, 7 cores were collected (5 for flux experiments and 2 for immediate sectioning). Cores were collected during a ~1–2 h period, capped immediately along with the overlying water, and stored upright on ice during transport within ~1 h to a refrigerated room (4 °C) where they were kept in the dark until either sectioning or flux experiments.

In addition to the cores, 20 L of bottom water was collected during each sampling trip from 0.5 m above the sediment surface with a Van Dorn sampler and stored in 10-L acid washed Cubitainers®. This bottom water was quickly (~1 h) returned to a refrigerated room, where it was kept until use in the flux experiments. Bottom water (10 mL) was subsampled, filtered (0.45 µm), and frozen for phosphate (PO_4_^3−^), ammonium (NH_4_^+^), and nitrate + nitrite (NO_3_^−^+ NO_2_^−^) concentration analysis at Wetland Biogeochemistry Analytical Services (WBAS, Louisiana State University). Standard US EPA methods were applied for all nutrient analyses (O'Dell, 1993; Zhang et al., 1997; Zimmermann and Keefe, 1997).

### 2.3 Flux experiments

Flux experiments began within 2 weeks of sediment core collection, and 3 separate experiments were conducted to test the impact of Phoslock® and redox conditions on sediment-water nutrient fluxes. Experiment 1, with the first collection of cores, tested nutrient fluxes in unaltered sediment under conditions of bottom water oxia and anoxia. Experiment 2, with the second collection of cores, considered the effect of Phoslock treatment with oxic bottom waters. Experiment 3, with the third collection of cores, considered the effect of Phoslock treatment with anoxic bottom waters. The use of unaltered sediment as controls in experiments 2 and 3, allowed us to test the relevant research question while minimizing the influence of seasonal differences between late winter and early spring. For each experiment, fluxes were generally measured multiple times per core after the replacement of overlying water with fresh bottom water, and these separate flux measurements are referred to as ‘trials’. With the combination of replicate cores and trials, multiple pseudo-replicates were established for each treatment condition.

Prior to the first trial of each experiment, cores and bottom water were allowed to equilibrate to room temperature (19–20 °C), and overlying water was carefully removed from each core via syphon and replaced with 150 mL of bottom water. This volume was chosen to be small enough to be responsive to benthic nutrient fluxes, yet large enough to not be substantially affected by the removal of 9–10 mL sample volume. Deviations from the following procedure when Phoslock® was dosed are described in the next paragraph. Humidified air or nitrogen gas (depending on oxic vs. anoxic treatment) was bubbled through the overlying water at a metered flow rate sufficient to ensure mixing of the water column without re-suspending sediment, using J-shaped steel HPLC tubing which lay just above the sediment water interface. In this way, the bubbles flowed gently upwards from the sediment-water interface, mixing the water column without resuspending the sediment. This rate was tested and confirmed with a dye-tracer experiment. All cores were wrapped with aluminum foil throughout the experiment to keep the sediments and overlying water dark, and the temperature of the room, measured multiple times each day, was consistently 19–20 °C. Overlying water samples were taken at known time intervals, with the initial sample taken at the start of gas bubbling, over a period of 1–5 days. Samples were filtered (0.45 µm, 9–10 mL), and frozen prior to analysis for PO_4_^3−^, NH_4_^+^, and NO_3_^−^ + NO_2_^−^ at WBAS. After removing an aliquot of overlying water, the same volume of bottom water was added to each core, maintaining an overlying water volume of 150 mL. Nutrient concentrations in this bottom water were measured for each sampling day, and used to correct the overlying water concentration for the effect of replacement. For Experiment 1, all 4 cores were run under oxic conditions for the first trial, after which the bottom water was replaced and 2 cores were run under oxic conditions and 2 cores under anoxic conditions for the second trial.

For Experiments 2 and 3, three cores were treated with Phoslock® and two cores remained untreated as the control, and the experiments continued over the course of 3 trials. Sediment cores treated with Phoslock® were dosed prior to the start of the first trial only. Phoslock® was provided by SePRO Corporation (SePRO Research and Technology Campus, Whitakers North Carolina, USA). The mass of Phoslock® added was determined from the proposed addition to Jordan Lake given by SePRO at 1.8 m^3^•ha^−1^ scaled to the surface area of the core (West Bishop, *personal communication*). This amounted to 0.5 g Phoslock® per 4.7 cm (id) diameter core, creating a Phoslock® layer approximately 1–2 mm thick. This dosage is in the range of dosage rates used in previous studies (e.g., 100–200% the dosage used by Gibbs et al., 2011 and ~25% of the dosage rate of Reitzel et al., 2013). When Phoslock® was dosed, 140 mL of bottom water was added to the core instead of the full 150 mL. Then, 5 mL overlying water was added to a scintillation vial containing 0.5 g Phoslock®, which was poured over the core, followed by 5 mL of overlying water as a rinse. Our application method was intended to create an even distribution of Phoslock® across the sediment-water interface. After Phoslock® had settled (5–6 h), gas flow was turned on for all Phoslock® and control cores, and the initial sample of the experiment was taken.

The PO_4_^3−^ flux was calculated from the slope of corrected PO_4_^3−^ concentration vs. time multiplied by the volume of overlying water and divided by the surface area of the sediment. In the first trial of anoxic treatments (Experiment 1 - trial 2, or Experiment 3 – trial 1), PO_4_^3−^ fluxes appeared to be delayed by ~20 h, which was likely caused by the slow transition of surface sediments from oxic to anoxic. More precisely, we presume this time-lag was caused by the slow reduction of Fe(III) to Fe(II), given our observation of a change in color from red to gray at the sediment surface. This is consistent with recent observations from Lake Erie, where PO_4_^3−^ release was not observed under hypoxic conditions, but instead was observed between 12 and 24 h following the onset of anoxic conditions (Anderson et al., 2021). Due to this suppression of PO_4_^3−^ fluxes at the start of the anoxic trials, PO_4_^3−^ fluxes were only calculated after this 20-hour time window had passed, once the PO_4_^3−^ concentration started increasing. The NH_4_^+^ flux was calculated in a similar manner. However, because we observed a non-negligible accumulation of NH_4_^+^ in the overlying water at longer incubation times (which artificially depresses measured and calculated sediment-water fluxes), we calculated NH_4_^+^ fluxes using the first 24–40 h of each trial.

### 2.4 Core sectioning and porewater chemistry

Sediment cores selected for immediate sectioning were processed within 1 week of retrieval from Jordan Lake, while those used in flux experiments were sectioned 1–3 days following the termination of the flux experiment. Prior to core sectioning, overlying water was carefully removed via syphon, and cores were extruded at 1-cm intervals for the top 3 cm, followed by 3-cm intervals for the remainder of the core. Extruded sediment was collected in clean 65-mL centrifuge tubes, cooled in a refrigerator (4 °C), then centrifuged at 4 °C to separate porewater and sediment. Porewater was passed through a 0.45-µm syringe filter and frozen until PO_4_^3−^, NH_4_^+^, and NO_3_^−^ + NO_2_^−^ analysis at WBAS. As we did not expect to find NO_3_^−^ + NO_2_^−^ below 3 cm, we analyzed NO_3_^−^ + NO_2_^−^ for the top 3 cm only. Care was taken throughout the process to minimize the introduction of oxygen prior to filtering, which could decrease the amount of PO_4_^3−^ by forming particulate Fe(III) complexes. Samples for PO_4_^3−^ were acidified prior to analysis, which would free any PO_4_^3−^ adsorbed on Fe(III) in the post-filtered sample.

## 3 Results

### 3.1 Flux experiments

**Fig. 2:**
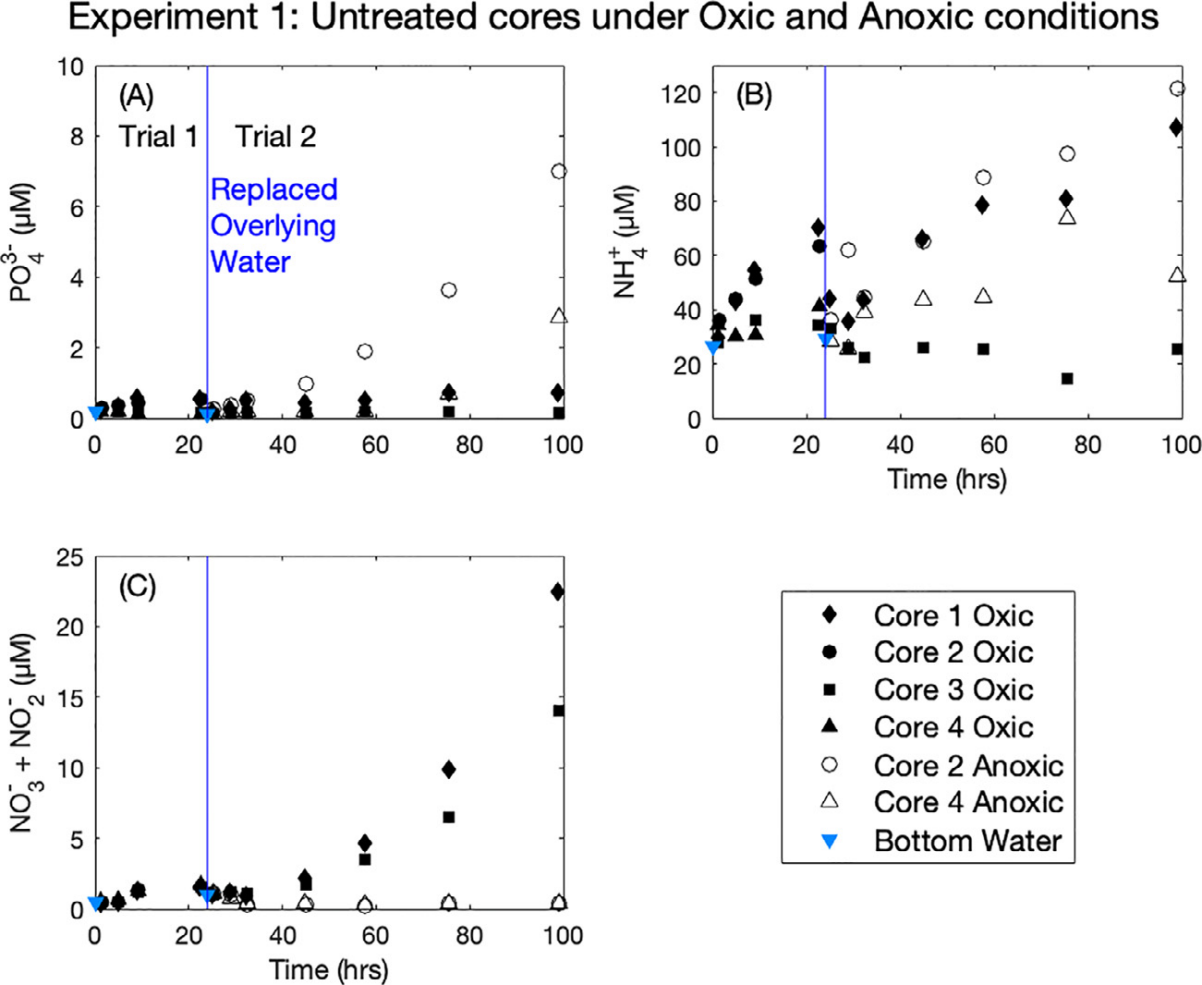
Corrected overlying water nutrient concentrations for Experiment 1 separated by analyte (PO_4_^3−^ (A), NH_4_^+^ (B), and NO_3_^−^ + NO_2_ (C)). Coloring is consistent for all figures, with black colors denoting untreated control cores. Filled symbols indicate oxic conditions and open symbols indicate anoxic conditions. Each core has a unique symbology, for example Core 2 is a circle under both oxic (filled) and anoxic (open) conditions. Vertical blue lines in all graphs indicate the replacement of bottom water and the start of the subsequent trial. Bottom waters were subsampled for nutrient analysis prior to each replacement of overlaying water (blue triangles).

In Experiment 1 (Oxia vs. Anoxia in untreated lake sediment), we found that PO_4_^3−^ did not appreciably increase with time for untreated cores under oxic conditions (Fig. 2, (A), filled symbols) for either trial, however under anoxic conditions there was a measurable release of PO_4_^3−^ from the sediment (Fig. 2, (A), open symbols). We observed a ~20-hour delay of PO_4_^3−^ release for Cores 2 and 4, presumably due to the time required to reduce Fe(III) to Fe(II) on the sediment surface as the cores transitioned from oxic to anoxic conditions (Anderson et al., 2021). We observed variable response of NH_4_^+^ under oxic conditions, with concentrations increasing during the first Trial by large amounts for Core 1 and Core 2 and smaller amounts for Core 3 and Core 4. Increases in NH_4_^+^ continued for Core 1 in the second trial, while reductions in NH_4_^+^ were observed for Core 3 (Fig. 2, (B), filled symbols). Under anoxic conditions, overlying NH_4_^+^increased the most for Core 2, with a moderate increase observed for Core 4 (Fig. 2, (B), open symbols). Changes in NO_3_^−^ + NO_2_^−^ were not observed under anoxic conditions (Fig. 2, (C), open symbols) while increases were observed under oxic conditions, especially in Trial 2 after ~48 h had passed (Fig. 2, (C), filled symbols).

**Fig. 3:**
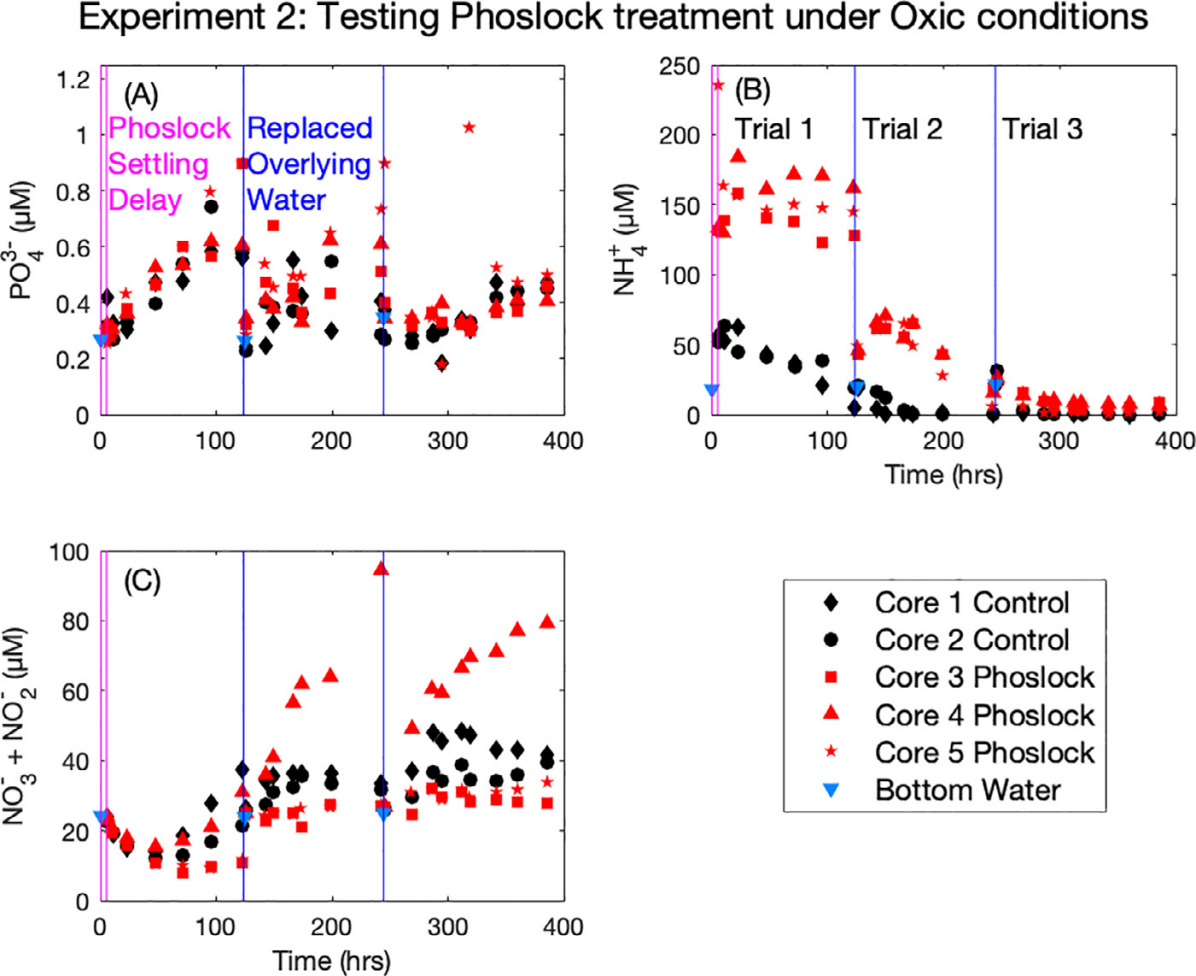
Corrected overlying water nutrient concentrations for Experiment 2 separated by analyte (PO_4_^3−^ (A), NH_4_^+^ (B), and NO_3_^−^ + NO_2_ (C)). Coloring is consistent for all figures, with black colors denoting untreated control cores, and red colors denoting Phoslock® treatment. Filled symbols indicate oxic conditions. Vertical pink lines indicate the delay between the first overlaying water replacement and Phoslock® addition, and the start of air bubbling and subsequent first sample, about 5 h. Vertical blue lines indicate the replacement of bottom water and the start of the subsequent trial. Bottom waters were subsampled for nutrient analysis prior to each replacement of overlaying water (blue triangles).

In Experiment 2 (Phoslock® treatment under oxic conditions), we found that the overlying water PO_4_^3−^ concentration did not appreciably change with time under oxic conditions for both Phoslock® treatment and the control, for all three trials (Fig. 3, (A), note the y-axis scale). For both NH_4_^+^ and NO_3_^−^+ NO_2_^−^ we found very strong differences between trials (Fig. 3, (B) and (C)). NH_4_^+^ concentrations at the first timepoint were elevated in the Phoslock® treatments, and remained slightly elevated relative to bottom water even after overlying water was replaced for the second trial (Fig. 3, (B), red symbols and blue triangle). Despite the elevated initial concentration, we observed NH_4_^+^ uptake in each subsequent trial for both Phoslock® and control, which we attribute to apparent nitrification (Fig. 3, (B)). Bottom waters collected for Experiment 2 were elevated in NO_3_^−^ + NO_2_^−^ compared to bottom waters for Experiments 1 and 3 (Fig. 3, (C), blue triangles). This concentration gradient fueled sediment uptake of NO_3_^−^ and NO_2_^−^ during the first half of Trial 1 for both Phoslock® and control (Fig. 3, (C)). In subsequent trials, overlying water NO_3_^−^+ NO_2_^−^ concentrations increased with time (Fig. 3, (C)), most strongly for the Phoslock® treated core 4.

**Fig. 4:**
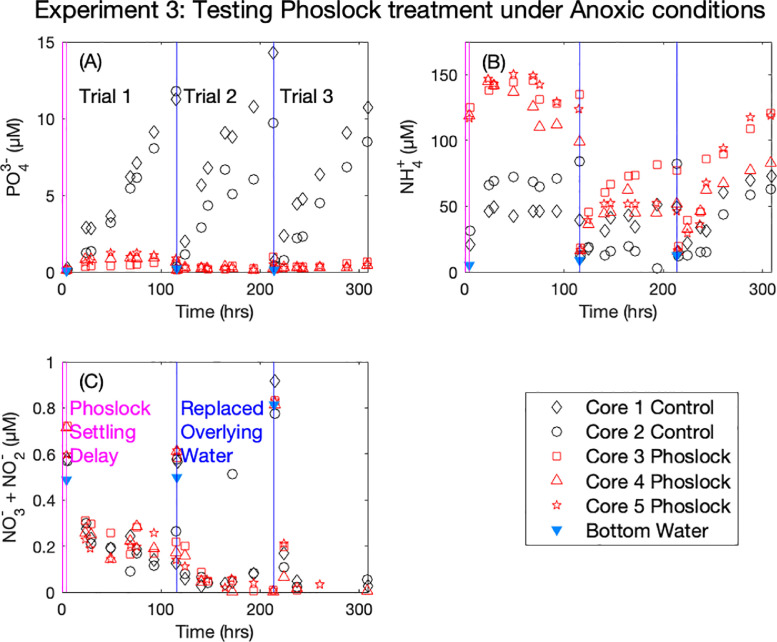
Corrected overlying water nutrient concentrations for Experiment 3 separated by analyte (PO_4_^3−^ (A), NH_4_^+^ (B), and NO_3_^−^ + NO_2_ (C)). Coloring is consistent for all figures, with black colors denoting untreated control cores, and red colors denoting Phoslock® treatment. Open symbols indicate anoxic conditions. Vertical pink lines indicate the delay between the first overlaying water replacement and Phoslock® addition, and the start of N_2_ bubbling and subsequent first sample, about 5 h. Vertical blue lines indicate the replacement of bottom water and the start of the subsequent trial. Bottom waters were subsampled for nutrient analysis prior to each replacement of overlaying water (blue triangles).

In Experiment 3 (Phoslock® treatment under anoxic conditions), we found that under anoxic conditions untreated sediment cores exhibited a measurable PO_4_^3−^ concentration increase (Fig. 4, (A), black symbols), while Phoslock® treatment prevented such PO_4_^3−^ release under otherwise identical conditions (Fig. 4, (A), red symbols). We again observed an increase in NH_4_^+^ concentration for the first timepoint of Trial 1 with Phoslock® treatment (Fig. 4, (B), red symbols). For untreated cores, as well as Phoslock® treated cores after Trial 1, NH_4_^+^ concentrations increased in the overlying water with time, although this trend was weaker for Core 2 in Trial 2 (Fig. 4, (B)). NO_3_^−^ + NO_2_^−^ concentrations remained low for all treatments and cores, consistent with the suppression of nitrification under anoxia (Fig. 4, (C), note the y-axis scale). We are cautious about interpreting the relative impact of Phoslock® on the nitrogen cycle under either oxic or anoxic conditions, because the potential impact of the large and sudden increase in NH_4_^+^ concentration (130–240 µM) is enhanced by our experimental choice of a batch incubation setup. However, previously reported flow through incubations may have missed this NH_4_^+^ release due to the exchange of water between Phoslock® dosing and the start of the experiment (Gibbs et al., 2011). Due to our artificially small overlaying water volume (150 mL vs ~7.5 L for the average water column depth over the same area in Jordan Lake), the concentration increases in NH_4_^+^ due to Phoslock® treatment would be much greater in our experimental setup than it would if applied to Jordan Lake. This, in turn, could have lingering impacts on NH_4_^+^ and NO_3_^−^+ NO_2_^−^ observations in subsequent Trials either by artificially affecting nitrification and subsequent denitrification rates, or by artificially increasing the porewater concentrations in the upper few centimeters of the core.

### 3.2 Core profiles and bottom water chemistry

**Fig. 5:**
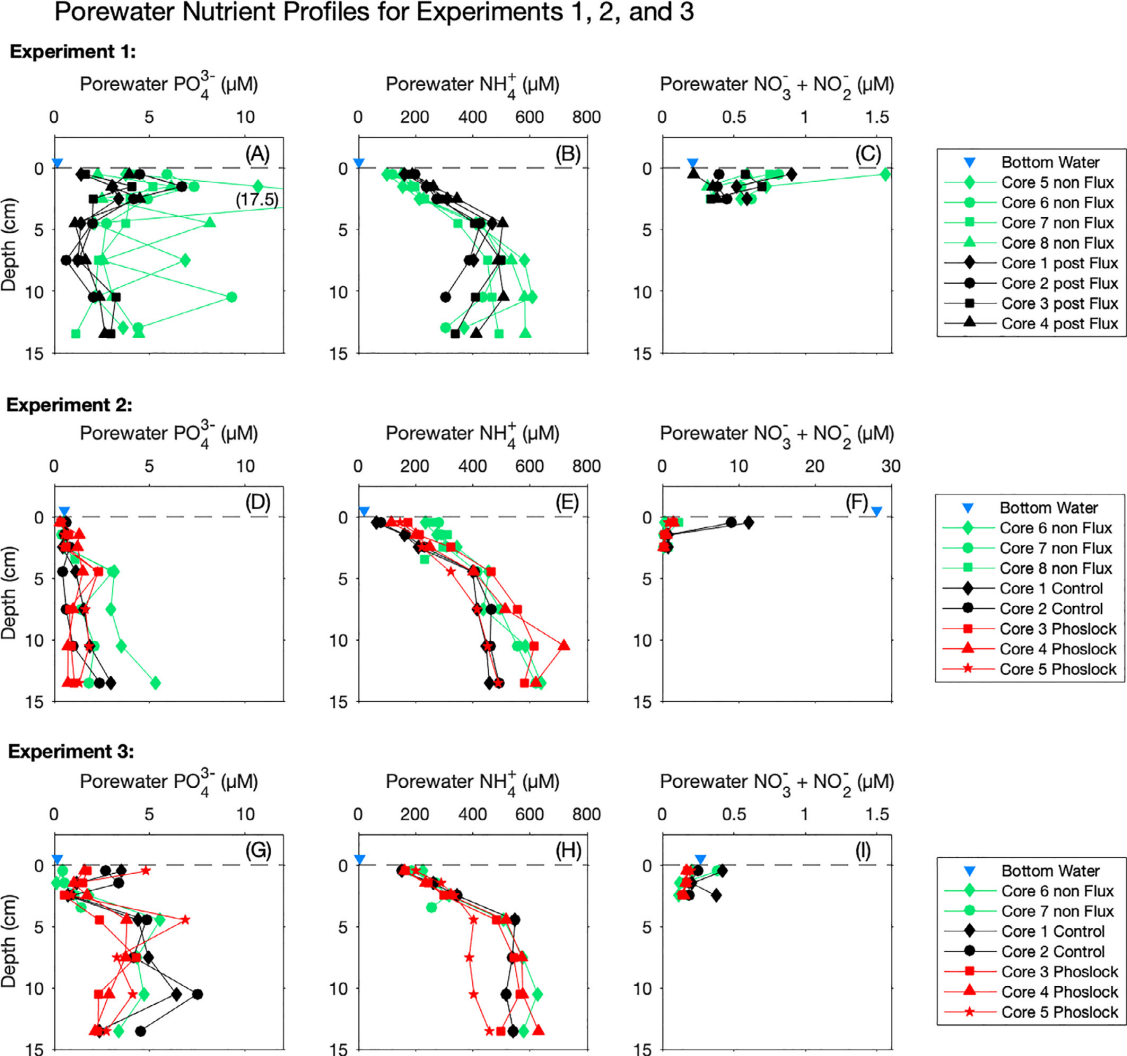
Porewater nutrient profiles of sectioned cores, separated by analyte (PO_4_^3−^, NH_4_^+^, and NO_3_^−^+ NO_2_^−^) and Experiment (1, 2, and 3). non Flux (Green): Cores which were sectioned upon collection. Control (black): untreated cores which underwent flux incubations prior to sectioning. Phoslock (red): Phoslock® treated cores which underwent flux incubations prior to sectioning. Points are placed at the midpoint depth of each fraction. Many cores were longer than 15 cm, however only the first 15 cm are presented here. Bottom waters (blue triangle) are artificially given a depth of “−0.5 cm”. The sediment-water interface is indicated with a horizontal dashed line. Scales are consistent for each analyte, with the exception of (F), which has a different x-axis scale than (C) or (I).

Our approach to quantifying sediment-water fluxes requires that porewater PO_4_^3−^ and NH_4_^+^ concentrations remained stable for the 0–2 weeks prior to the start of the flux experiment (storage at 4 °C), and for the 5–15-day duration of the flux experiments (room temperature). In general, porewater nutrient profiles for cores that were sectioned shortly after collection (Fig. 5 all charts, non Flux, green) were similar to profiles of cores that were sectioned after the completion of the flux experiments (Fig. 5 all charts, black and red), however there are some important deviations to point out. For each treatment (non Flux, post Flux, Control, and Phoslock) and experiment, we conducted ANOVA (RStudio) to compare the means. While the small sample size (*n* = 2–4) is an issue for this analysis, the results for the top 5 cm can be found in the supporting information (Supplementary Figure S1). In Experiment 1, Core 5 non Flux showed elevated PO_4_^3−^ concentrations in the top three cm (Fig. 5, (A), green diamond), although the other three non Flux cores were much more similar to the post Flux cores (Fig. 5, (A)), and the differences were not significant (*p* > 0.1). In depths lower than the top three cm, Core 8 and Core 6 non Flux also had at least one elevated fraction (Fig. 5, (A), green circle and triangle), however it should be noted that the flux experiments would be most sensitive to the uppermost fraction PO_4_^3−^ concentrations, as these have the greatest influence on diffusive fluxes. Post Flux cores showed a small elevation in NH_4_^+^ concentration relative to non Flux cores (*p* < 0.05) in the uppermost fractions, and this trend was reversed at depths below 7 cm (Fig. 5, (B)). For NO_3_^−^ + NO_2_^−,^ all concentrations were low for both post Flux and non Flux cores (Fig. 5, (C)) although a slight elevation was observed at 0.5 cm for Core 5 non Flux (Fig. 5, (C), green diamond). For this experiment, a trend in NH_4_^+^ or NO_3_^−^ + NO_2_^−^ porewater concentrations based on Trial 2 oxic treatment (Core 1 and Core 3) vs anoxic treatment (Core 2 and Core 4) could not be established.

In Experiment 2, porewater PO_4_^3−^ concentrations were broadly similar between non Flux, control, and Phoslock® cores, especially through the top 5 cm (Fig. 5, (D)). Although Core 6 non Flux was elevated relative to the others at depths (Fig. 5, (D), green diamond), all cores exhibited lower PO_4_^3−^ concentrations in the upper 5 cm relative to those cores sectioned for Experiment 1 (Fig. 5, (A) vs. (D)). For NH_4_^+^, we observed moderately higher concentrations in the top 3 cm for non Flux cores (*p* < 0.05), and Phoslock® cores also moderately elevated over Control cores (Fig. 5, (E)), although these trends did not continue with depths. Although studies with different experimental setups have associated elevated porewater NH_4_^+^ concentrations with reductions in nitrification rates due to Phoslock® treatment (Lin et al., 2017; Song et al., 2020), we are unable to say whether the elevated porewater NH_4_^+^ that we observed was due to Phoslock® itself, or due to the pulse of NH_4_^+^ to the overlaying water upon Phoslock® dispersal. The Control cores had much higher NO_3_^−^ + NO_2_^−^ concentrations at 0.5 cm (*p* < 0.001) than either the non Flux or Phoslock® cores (Fig. 5, (F), black), which is likely related to the lower NH_4_^+^ values due to increased nitrification under the oxic conditions.

In Experiment 3, PO_4_^3−^ concentrations in Control and Phoslock® cores were elevated relative to the non Flux cores in the first 0.5 cm fraction, however this difference did not continue down core (Fig. 5, (G), black and red vs. green), and it was also not significant (*p* > 0.1). However, because only data from the top 5 cm were available for Core 6 non Flux, we only have one full profile of a non Flux core for this experiment. NH_4_^+^ profiles were broadly similar across treatments (Fig. 5, (H), *p* > 0.1), however Core 5 Phoslock® had somewhat lower concentrations below 5 cm (Fig. 5, (H), red star). NO_3_^−^ + NO_2_^−^ concentrations were low regardless of treatment (Fig. 5(I)). For all three experiments, bottom water concentrations were generally lower than the first porewater 0.5 cm fraction for all analytes, with the exception of Experiment 2, in which the bottom water had the same PO_4_^3-−^ concentration as most porewater 0.5 cm fractions (Fig. 5, (D), blue triangle) and NO_3_^−^ + NO_2_^−^was much higher than all porewater 0.5 cm fractions including the Control cores (Fig. 5, (F), blue triangle).

## 4 Discussion

Prior experimental determinations of the impact of Phoslock® on PO_4_^3−^ flux used cores packed with homogenized sediment, with the intention of reducing variability between cores (Egemose et al., 2010; Gibbs et al., 2011; Reitzel et al., 2013). However, homogenization also has the unintended effect of blurring the gradients near the sediment-water interface, and affects sediment physico-chemical properties, thereby artificially changing diffusive fluxes. Hence, homogenized cores are considered less representative of the natural lake sediments than intact cores. Our strategy of using some cores only for sectioning allows us to use intact cores and also address the variability and the impact of the experimental design on the results. We did so by conducting a two-factor ANOVA test (RStudio, aov Condition + Trial, type III) considering the impact of Trial number and condition (oxic control, oxic Phoslock®, anoxic control, anoxic Phoslock®) on sediment-water fluxes, which showed that mean PO_4_^3−^ fluxes were not significantly different between Trials (*p*>>0.05), despite significant differences based on condition (*p*<< 0.05). Given the visible similarity of our porewater profiles, and the lack of significant relationship between PO_4_^3−^ flux and trial number, we can conclude that our experimental design was reasonably effective at operating under steady-state conditions, allowing us to consider our pseudo-replicates as true replicates.

**Fig. 6:**
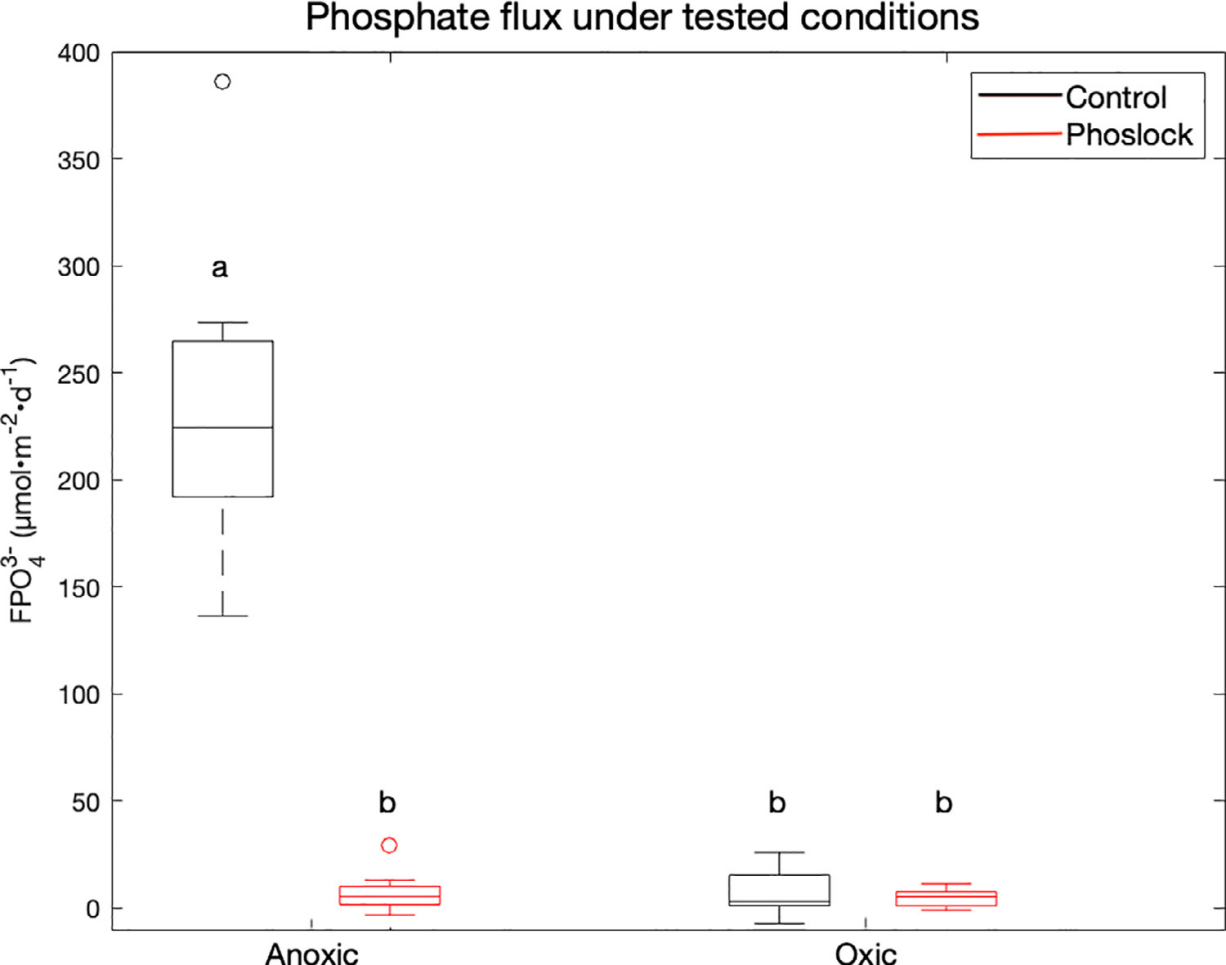
Fluxes for PO_4_^3−^ in µmol•m^−2^•d^−1^, separated by treatment (Control, black and Phoslock®, red) and condition (Anoxic, left and Oxic, right). Flux calculations from all three experiments and trials are pooled.

The primary goal of this study was to establish the relative efficacy of Phoslock® amendment and natural sedimentary iron under oxic and anoxic bottom water conditions, in clay-rich sediments. We found that, when the overlying water was oxygenated, PO_4_^3−^ flux was not statistically different (*p* >> 0.05) between the control (7.0 ± 11.4 µmol•m^−2^•d^−1^) and Phoslock® treatments (4.5 ± 4.3 µmol•m^−2^•d^−1^, Fig. 6, Oxic). These results are in contrast to a prior study in Lake Rotorua, a volcanic lake with very high internal P in New Zealand, where it was found that Phoslock® amendment could increase sediment uptake of DRP (dissolved reactive phosphate) under oxic conditions to approximately −12 mg•m^−2^•d^−1^ from approximately −2.5 mg•m^−2^•d^−1^ for the control (Gibbs et al., 2011). Our results are also in contrast with results from Lake Langesø, which indicated that Phoslock could improve P uptake from −44 µmol•m^−2^•d^−1^ (control) to 51 µmol•m^−2^•d^−1^. This highlights the importance of sediment and lake type when assessing the potential efficacy of Phoslock®. Jordan Lake's bottom waters often become suboxic in mid-summer, and under anoxic conditions, we found that Phoslock® treatment effectively decreased sediment PO_4_^3−^ fluxes (7.5 ± 9.5 µmol•m^−2^•d^−1^) relative to untreated sediment (236 ± 74 µmol•m^−2^•d^−1^, Fig. 6, Anoxic). This finding is similar to the results reported from Lake Rotorua (Gibbs et al., 2011), where a DRP flux of −8 mg•m^−2^•d^−1^ was observed with Phoslock® treatment, and 27 mg•m^−2^•d^−1^ flux for the control. The iron-rich Jordan Lake sediment creates a natural barrier to PO_4_^3−^ release, but only under oxic conditions, as PO_4_^3−^ bonds nearly irreversibly with Fe(III). This natural barrier to PO_4_^3−^ release was active during our study, as seen in the statistically indistinguishable PO_4_^3−^ fluxes between the Phoslock® and oxic control treatments (Fig. 6, *p*>>0.05). In other words, unamended Jordan Lake sediments are just as effective at reducing PO_4_^3−^ fluxes as sediments with Phoslock® treatment, provided that the bottom water is oxygenated. Jordan Lake experiences water quality issues throughout the year, even when the bottom water is oxygenated during the fall through spring (Cain, 2017; Wiltsie et al., 2018). Our results suggest that Phoslock® treatment will not mitigate these water quality issues when the water column is mixed, and may only be effective at reducing the intensity summer algal blooms.

**Fig. 7:**
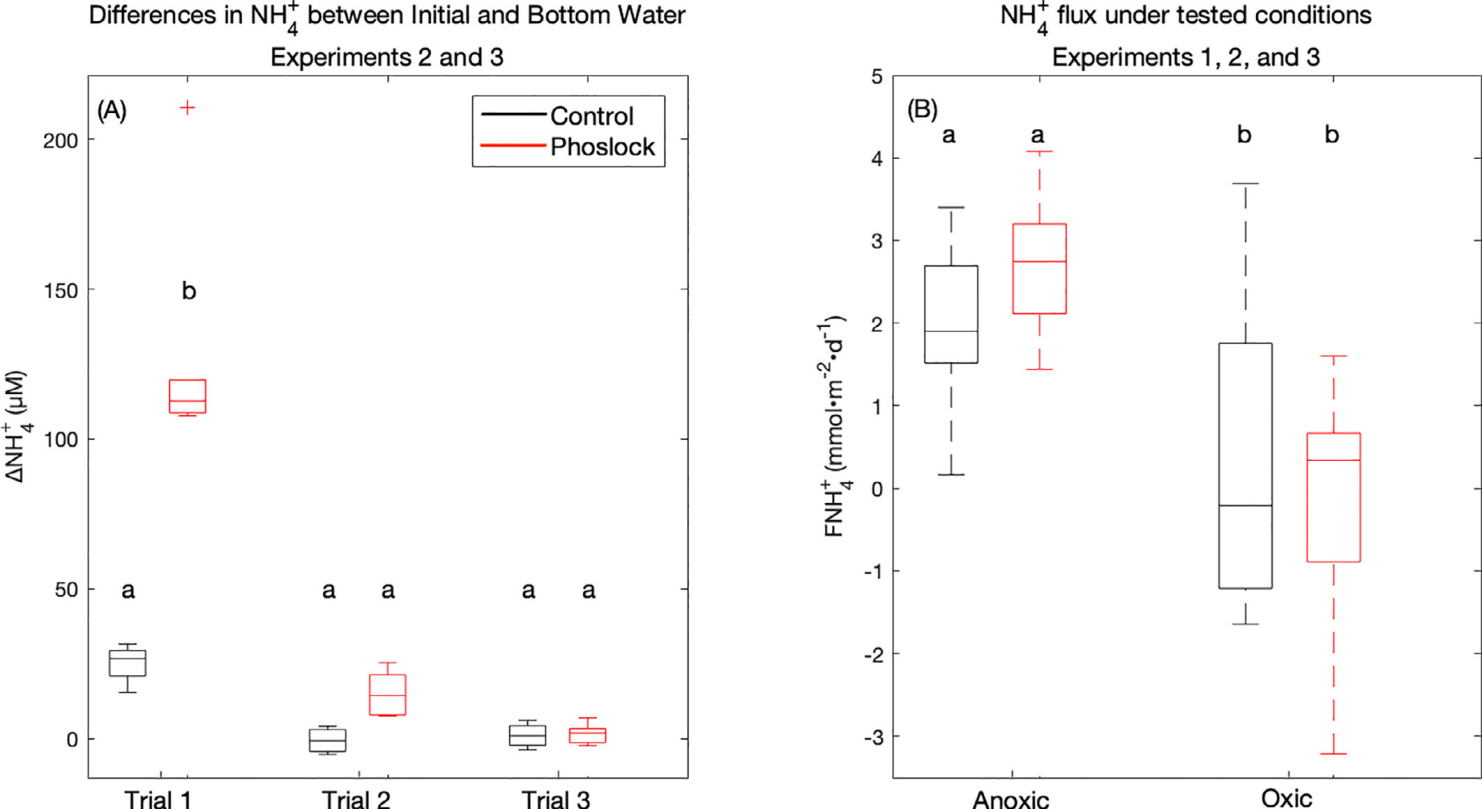
(A) The difference between the NH_4_^+^ concentration of the first time point (Initial) subtracted from the bottom water concentration at the start of each trial. Data is shown for Experiments 2 and 3, and separated by Trial (1, 2, or 3) and treatment (Control, black or Phoslock®, red), but is pooled by condition (Anoxic and Oxic). (B) The flux of NH_4_^+^ (mmol•m^−2^•d^−1^) calculated for each treatment (Control, black or Phoslock®, red) and condition (Anoxic, left and Oxic, right), but pooled by Trial.

We also observed an unexpected and large release of NH_4_^+^ directly from Phoslock®. This NH_4_^+^ release was enough to increase the NH_4_^+^ concentration of the 150 mL overlying water by 129 ± 40 µM above bottom-water levels for the first trial (Fig. 7, (A), Trial 1 Red). This NH_4_^+^ release from Phoslock® could be a significant input of labile N to Jordan Lake. Assuming a 4.3 m water column (the average depth of Jordan Lake), the NH_4_^+^released from Phoslock® amounts to an increase in lake NH_4_^+^ concentration of 2.6 ± 0.8 μM. As the observed bottom water NH_4_^+^ concentrations ranged from 1.22 to 19.4 μM (Fig. 5, (B), (E), and (H), blue), this increase represents a roughly 10 to 275% increase in whole lake water column NH_4_^+^ concentrations. Using the NH_4_^+^ benthic flux for the anoxic controls (2.0 ± 1.0 mmol NH_4_^+^m^−2^ d^−1^; Fig. 7, (B) anoxic black), we can estimate that the sediment contribution of NH_4_^+^ increases water column concentrations by roughly 0.48 ± 0.24 µM d^−1^. In this case, the anoxic control NH_4_^+^ flux is used because the NH_4_^+^ flux is less affected by either nitrification or the pulse of NH_4_^+^ from Phoslock® treatment. Thus, Phoslock® could contribute the equivalent of roughly 2.5–14 days (median 5.5 days) of sediment NH_4_^+^ loading within minutes to hours of its application (although this estimate is calculated using our experimental results, from a temperature of 19–20 °C).

A small increase in NH_4_^+^ was also observed in the control for the first trial (Fig. 7, (A) Trial 1, black), which was likely a result of the initial sample for this trial being collected 5–6 h after the addition of bottom water, to allow for Phoslock® settling, and could represent a 5–6-hour NH_4_^+^ flux. This factor was not present in subsequent trials (Fig. 5, (A) Trial 2 and Trial 3, black) which did not have such a delay, which indicates that our bottom water addition process was minimally disruptive to the sediment-water interface. As Phoslock® was only added prior to the first trial and overlying waters were replaced between trials, the impact of Phoslock® on the initial NH_4_^+^ concentration was greatly reduced for Trial 2 and eliminated for Trial 3 (Fig. 7, (A) Trial 2 and Trial 3, red).

Despite the increase in initial NH_4_^+^ concentrations, we saw sediment uptake of NH_4_^+^ under oxic conditions relative to anoxic conditions, and this difference persisted across Control and Phoslock® treatments (Fig. 7, (B)), suggesting rapid nitrification of NH_4_^+^ (Fig. 3, (B) and (C)). While the reduction in overlying water NH_4_^+^ throughout Experiment 2 suggests that this input of NH_4_^+^ could be rapidly cycled in an oxygenated Jordan Lake (Fig. 3, (B), red), there is reason to suspect that Phoslock® treatment could reduce the ability of the sediments to remove excess NH_4_^+^ through nitrification/denitrification processes relative to untreated lake sediment. Phoslock® has been shown to decrease the oxygen penetration depth in sediments, potentially into the Phoslock® layer itself for larger Phoslock® treatments (Vopel et al., 2008), which could decrease nitrification rates which occur in the thin oxygenated zone of sediments. Extended Phoslock® treatment has been linked to a reduction in nitrification rates, estimated either through increased porewater NH_4_^+^ (Song et al., 2020) or through reductions in abundance of archaeal ammonia-oxidizers (Lin et al., 2017). In our study, Phoslock® treated cores also showed elevated NH_4_^+^ and reduced NO_3_^−^ + NO_2_^−^ porewater concentrations relative to the Control cores (Fig. 5, (E) and (F), red vs. black), which provides some evidence for this effect. However, the results of Experiment 2 (Phoslock® under oxic conditions) offer mixed evidence of reduced nitrification with Phoslock®, as Phoslock® treated Cores 3 and 5 show reduced water column NO_3_^−^ + NO_2_^−^ relative to the Control Cores 1 and 2, while Core 4 Phoslock® shows elevated NO_3_^−^ + NO_2_^−^ (Fig. 3, (C)). As the batch incubation heightened the impact of the NH_4_^+^ input from Phoslock®, and as we did not measure N_2_ or quantify nitrification or denitrification rates, these results can only provide limited qualitative, rather than quantitative, support for Phoslock® reducing nitrification rates in lake sediments.

Our study is not the first to identify Phoslock® as a potential source of NH_4_^+^. For example, van Oosterhout et al. found that Phoslock® released NH_4_^+^ into nanopore water at a rate of 223 mg kg^−1^ Phoslock® (van Oosterhout and Lürling, 2013). Reitzel et al. reported that 4 grams of Phoslock® could release 10.3 mmol NH_4_^+^ m^−2^ when dispersed in Milli-Q water. However, they did not observe a corresponding increase in NH_4_^+^ when dispersed in their Lake Langesø water during their laboratory incubations, which they attributed to the lake's high alkalinity (Reitzel et al., 2013). Thus, the NH_4_^+^ release from Phoslock® may vary with lake water chemistry (especially alkalinity). However, our study shows that substantial NH_4_^+^ release is likely in Jordan Lake, and should be considered when deciding whether Phoslock® application is appropriate. This is especially true because as a reservoir, Jordan Lake is connected to the Cape Fear river and is upstream of the Cape Fear estuary, a potentially more N-limited system (Burkholder et al., 2006; Paerl, 2009). Simultaneously increasing N while effectively managing summertime P could have unintended consequences for Jordan Lake's N filtration ecosystem service (Finlay et al., 2013).

Our experiments did not fully address the reasons for NH_4_^+^ release from Phoslock®, but other studies have indicated that low alkalinity (Reitzel et al., 2013) could contribute to the release of NH_4_^+^, potentially due to an increase in the Phoslock® clay dispersion in soft water vs. hard waters with high Ca^2+^ concentration (Reitzel et al., 2017). If clay dispersion is the reason for this NH_4_^+^ release, then it is reasonable to expect that lanthanum may also be released with Jordan Lake water, although we did not measure it. Lanthanum is released under variable pH conditions (Ross et al., 2008), when humic or fulvic acid content is high (Dithmer et al., 2016; Reitzel et al., 2017; Reitzel et al., 2013; Wang et al., 2016), or when alkalinity is low (Reitzel et al., 2017; Spears et al., 2013). Our observed large release of NH_4_^+^ suggests that the Phoslock® matrix might not be stable in Jordan Lake waters and might be a source of heavy metal pollutants, including lanthanum.

## 5 Conclusion

This study tested the efficacy of Phoslock® in reducing internal phosphate nutrient loading from iron-rich sediments, in comparison with simple bottom water oxygenation. We found that Phoslock® did not provide an additional benefit compared to untreated sediment under conditions of bottom-water oxia in Jordan Lake (representative of fall through spring conditions), a piedmont reservoir in central North Carolina. However, during stratified summertime periods when Jordan Lake bottom waters experience anoxia, our results show that Phoslock® is more effective at lowering sediment phosphate fluxes, compared with the anoxic control without Phoslock®. This study did not test the long-term effectiveness of Phoslock®, which may decrease as sediments bury the Phoslock® amended layer. We also found that Phoslock® itself can be a source of ammonium when dispersed in Jordan Lake water. Our experimental treatment of 500 mg of Phoslock® per core increased NH_4_^+^concentration by 129 ± 40 µM in our 150 mL overlying waters, an equivalent to roughly 5.5 days of estimated sediment NH_4_^+^ loadings from untreated sediment. This NH_4_ release could exacerbate issues of N export to downstream areas associated with only focusing on P management strategies.

## Declaration of Competing Interest

None.

## Appendix Supplementary materials

Image, application 1
