# Supplementary material for: The efficacy of Phoslock® in reducing internal phosphate loading varies with bottom water oxygenation
Source: Water Res X. 2021 Mar 5;11:100095. doi: 10.1016/j.wroa.2021.100095 (PMC7974025; doi:10.1016/j.wroa.2021.100095)
Supplement: Supplementary file 1 [file mmc1.docx]

**Supplementary Information: The efficacy of Phoslock® in reducing internal phosphate loading varies with bottom water oxygenation**

Mary Zeller, Marc Alperin


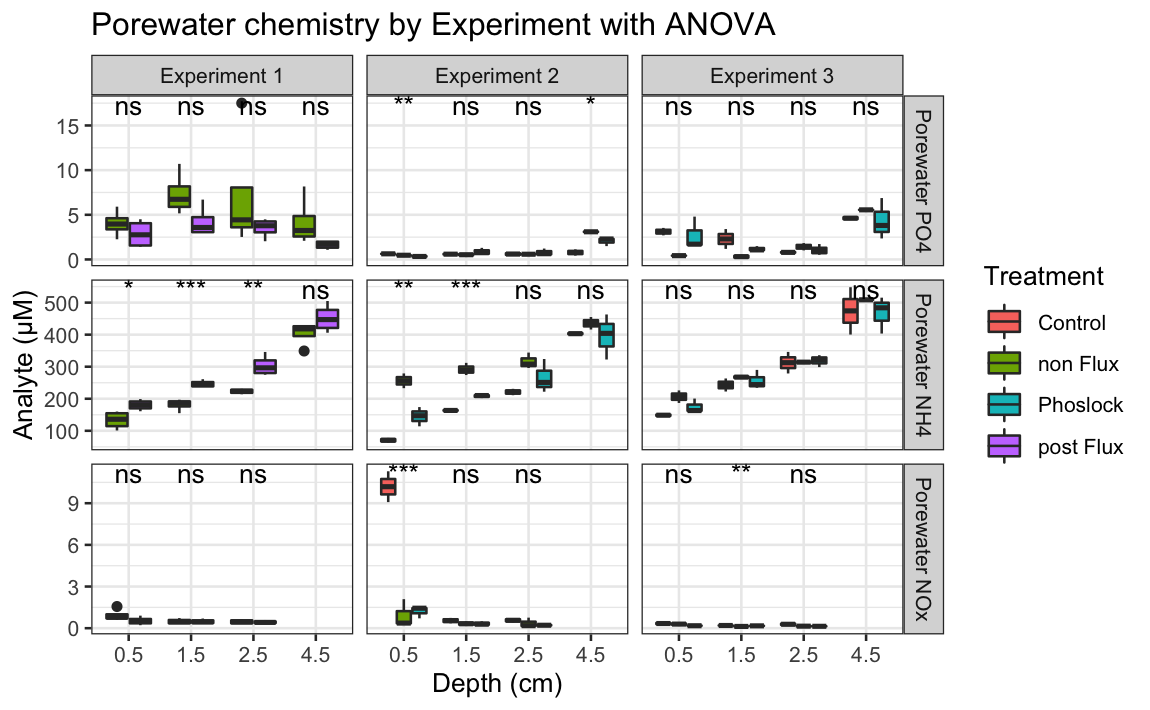


**Figure S1**: Porewater PO_4_^3-^ (top), NH_4_^+^ (middle) and NO_3_ + NO_2_ (bottom) concentrations for the various cores and experiments, and for each depth fraction above 5 cm. For Experiment 1 (left) non Flux (green, n = 4) and post Flux (purple, n = 4) values are provided. For Experiment 2 (middle), Control (orange, n = 2), non Flux (green, n = 3), and Phoslock (blue, n = 3) values are provided. For Experiment 3 (right), Control (orange, n = 2), non Flux (green, n = 2), and Phoslock (blue, n = 3) values are provided. Significance was tested with ANOVA in RStudio, ns = p > 0.1, · = 0.1 > p > 0.05 (no example in data), * = 0.05 > p > 0.01, ** = 0.01 > p > 0.001, *** = p < 0.001.
